# The Tetrodotoxin Binding Site Is within the Outer Vestibule of the Sodium Channel

**DOI:** 10.3390/md8020219

**Published:** 2010-02-01

**Authors:** Harry A. Fozzard, Gregory M. Lipkind

**Affiliations:** Department of Medicine, MC 6094, University of Chicago Hospitals, 5841 S Maryland Av. Chicago, IL 60637, USA; E-Mail: lipkind@uchicago.edu

**Keywords:** marine toxins, Na channels, molecular modeling

## Abstract

Tetrodotoxin and saxitoxin are small, compact asymmetrical marine toxins that block voltage-gated Na channels with high affinity and specificity. They enter the channel pore’s outer vestibule and bind to multiple residues that control permeation. Radiolabeled toxins were key contributors to channel protein purification and subsequent cloning. They also helped identify critical structural elements called P loops. Spacial organization of their mutation-identified interaction sites in molecular models has generated a molecular image of the TTX binding site in the outer vestibule and the critical permeation and selectivity features of this region. One site in the channel’s domain I P loop determines affinity differences in mammalian isoforms.

## 1. Introduction

Tetrodotoxin (TTX) and saxitoxin (STX)—guanidinium toxins—are potent and potentially lethal marine toxins with features that have been of great value to ion channel research. The two toxins are small molecules with similar structural properties and they block voltage-gated Na channels competitively. Because of their specificity for Na channels they allow separation of Na currents from other ionic currents in native cells. They are so called because they both have guanidinium moieties and are consequently both positively charged at neutral pH. The early history and chemistry of these toxins has been amply described in a review by Kao [1] and in a symposium report [2].

After Narahashi and colleagues [3,4] had shown that TTX blocked voltage-dependent Na currents, Hille [5] determined a number of important pharmacological characteristics. He confirmed that TTX and STX appeared to be completely specific for Na channels and bound with 1:1 stoichiometry. Hille [6] summarized the argument derived from functional data that TTX and STX bind in the external channel vestibule and block Na current by occluding the pore completely. The original points supporting this argument are:

1. Guanidinium ions are (slightly) permeable, implying that they can interact within the pore as deep as the selectivity filter. TTX and STX, although somewhat larger than guanidinium ions, have guanidinium moietie(s), plausibly allowing them to reach the narrowest part of the pore and interact with the selectivity filter, but block because they are too large to pass through [7].
2. Protons and the guanidinium toxins both have positive charges. Protons reduce both Na permeation and TTX block [5]. Their block is field-dependent; both appear to bind about one-third of the distance through the membrane electric field [7,8], presumably interacting with carboxylates involved in permeation.
3. The selectivity filter is presumed to contain carboxylates in order to facilitate dehydration of Na^+^ with minimal energy exchange and to permit it to distinguish between that ion and K^+^. TTX block is antagonized irreversibly by treatment with carboxyl-modifying agents [10–12].
4. TTX binding is antagonized by small cations, some of which are permeant [13,14]. For example, Ca^2+^ competes with TTX/STX and it blocks within the membrane electric field at the same apparent depth as TTX/STX.

These arguments are all inferential, but they have been valuable guides to experimental study of the toxin binding site.

The specificity of TTX for voltage-gated Na channels has been challenged by a suggestion that there is a cardiac Na channel with substantial permeability for Ca^2+^, that it is genetically different from the Nav family, and that it is nevertheless blocked by TTX [15,16]. This channel has not been cloned, so its properties remain unresolved. In addition there is an unconfirmed report that one isoform group of the T type Ca channel (Cav3) binds TTX and STX and is blocked with low toxin affinity [17].

## 2. The Role of Gating in TTX Block

The argument that TTX and STX achieve their block by occluding the outer pore was plausible but the evidence was circumstantial, and interference with channel gating is a logical alternative idea for the mechanism of block. TTX/STX block is somewhat use-dependent, which means that with repeated stimulation the block is enhanced about 3-fold [18]. The presence of use-dependence is reminiscent of the use-dependence of local anesthetic drugs, where drug-induced changes in gating are documented [19]. The most direct way to ask the question of gating effects of TTX/STX binding is measurement of gating currents. These are small capacitive currents generated by movement of charged amino acids within the membrane relative to the membrane resistive barrier by the channel’s voltage sensors (S4 segments), which are strongly positively charged [20]. Initial studies implied no effect of TTX on gating currents (e.g., [21]). However, subsequent careful measurements demonstrated that TTX can shift the voltage sensitivity of gating about 10 mV in the hyperpolarizing direction [22], with effects on gating kinetics that can be explained by the shift in voltage dependence. Other than this modest effect on the voltage range of gating, no other effects on gating currents are seen.

TTX carries one positive charge and STX carries two positive charges. To the extent that this charge enters the membrane electric field as it binds to its site in the pore ([23], but see [24,25]), it could have an electrostatic effect on the field acting on the voltage sensors. However, the effect of one positive charge would be very small, judging from the minimal effects of neutralization of the vestibule carboxylates by mutation on channel gating [26], and it is in the opposite direction to the experimentally observed voltage shift. Furthermore, STX with two positive charges has a smaller effect than TTX with its one charge. Heggeness and Starkus [22] proposed that TTX binding displaces a divalent ion (Ca or Mg) from within the pore, reducing the effect of its double charge, although the lesser effect of STX is difficult to explain by this mechanism. In any case, if this modest displacement of the voltage dependence of gating is involved in the mechanism of block, it could easily be removed by an appropriate hyperpolarization, and this does not occur. So it can be concluded that TTX and STX do not block by paralysis of gating.

## 3. Cloning of the Na Channel Family

The voltage-gated Na channel in excitable tissue is an intrinsic membrane protein that is present only in trace quantities. Since TTX and STX bind selectively with one toxin per channel, tritiated toxin has been successfully used to determine the density of Na channels in the membrane [27]. The very low concentration of Na channel protein in any biochemical preparation of membrane makes its purification difficult. Although the presence of channels in various fractions during purification could perhaps be determined by their electrical assay after incorporation in artificial bilayers, this is a very cumbersome method. However, the tritiated toxin can be used conveniently, and this was the approach for Na channel purification [28]. With purification, the necessary painstaking amino acid sequencing can be done, providing the initial amino acid sequence necessary for probes to select mRNA and allow synthesis of the cDNA sequence. The Numa laboratory in Kyoto [29] reported the deduced primary structure of the eel Na channel, and subsequently the primary sequences of the ~2,000 amino acids of the mammalian brain channels α-subunits. The homologous mammalian skeletal muscle isoform sequence was reported by Trimmer *et al*. [30] and George *et al*. [31]), and the cardiac isoform was soon reported soon thereafter [32,33]. Subsequently, many more Na channel isoforms have been cloned from mammalian peripheral nerve and from non-mammalian species. Goldin [34] has listed 26 mammalian, 11 other vertebrate, and 18 invertebrate Na channels clones. The large number of voltage-gated Na channel isoforms fits with variation of physiological and pharmacological properties of Na currents, and opens the door to correlation of structure with function.

## 4. Early Post-Cloning Insights

Cloning identified the ~2,000 amino acid residue primary sequence of the Na channel isoforms. Although only a few intrinsic membrane proteins had been structurally determined by that time, the patterns from them and from general rules of protein structure were helpful for predicting the Na channel secondary and tertiary structures [35]. Three features were important in the quest for locating the TTX binding site in this protein. (1) The protein contained four homologous sets (domains I–IV) of 6α-helical segments (S1–S6), each long enough to cross the membrane. (2) Between S5 and S6 in each domain is a short segment that appeared to fold into the membrane half-way (the P loop), and it contained regions that showed almost perfect homology across the 5–6 isoforms initially cloned (a Na channel “signature” sequence). (3). A mutation in that “signature sequence” resulted in loss of ion permeation without change in gating currents. These three features were important steps to locate the mouth of the pore, which had been proposed to be the TTX binding site.

The initial model of membrane topology of the Na channel by Noda *et al*. [29] was confounded by two areas difficult to characterize. Firstly, the S4 of each domain contained positively charged amino acid residues every third position, making it unstable within the hydrophobic interior of the membrane. It was initially proposed to form part of the pore lining because of its hydrophilic character [36]. However, it turned out to be the element that is responsive to membrane electric field and constituted a major part of the channel’s “voltage sensor”. Secondly, the region between S5 and S6 contained α-helical sequences that initially were thought to cross the membrane. Comparison to a recently cloned K channel helped to confirm that each domain of the channel contained only six transmembrane segments.

During examination of the sequences of the S5-S6 regions, Guy and Seetharamulu [37] proposed that they contain a hairpin element that folded partly into the membrane, initially called SS1 and SS2, and that this region formed part of the pore. Each of the four SS2 segments contained a sequence of 4–5 amino acid residues that was almost perfectly homologous among the 5–6 isoforms available at that time. This homology is what would be expected for a region that determined Na^+^ selectivity, the characteristic that defines Na channels. Each domain contained a different SS2 sequence, but the domain’s sequence was shared between isoforms. The SS2 segments included multiple carboxylates, which met the requirements of Hille’s suggestion [38] for a region that could dehydrate Na during its passage through the pore. The carboxylates could also be the residues interacting with trimethyloxonium [12,39], which altered current and TTX binding. This S5–S6 hairpin was named the “P loop”, and Guy [40] presciently presented a cartoon of the four P loops (one from each domain) forming the narrow part of the pore and interacting with TTX.

Following the suggestion from modeling studies that the carboxylates in SS2 of the P loop were part of the narrow pore, Pusch *et al*. [41] mutated one carboxylate (D387N in Nav1.2) and almost abolished Na current without affecting channel gating. The failure to alter gating, judged by the presence of normal gating currents, was strong evidence that the channel was folding properly and inserting in the membrane, even though little ionic current could be recorded. This result reinforced the idea that the P loop contains the narrow part of the pore and that it was a good candidate for the TTX binding site.

## 5. Mutations Affecting TTX Binding

**Figure 1:**
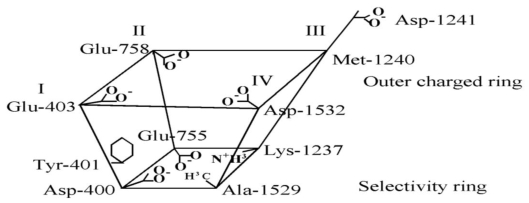
Schematic of the channel’s outer vestibule, showing some critical residues (Nav1.4 numbering).

The four P loops forming the pore’s outer vestibule contain multiple charged amino acids—six carboxylates and a lysine aligned into an inner ring (DEKA, with one residue from each domain) and an outer ring (EE(M/D)D) (Figure 1). Noda *et al*. [42] demonstrated that neutralization of the outer carboxyl of domain 1 in Nav1.2 (E387Q) abolished TTX block, with only modest changes in peak Na current magnitude and no change in gating. With this assurance that TTX did bind in this region, which was presumably the outer pore, Terlau *et al*. [43] showed that neutralization of each of seven of the charged residues had significant effects on TTX block, some by factors of 10^4^. Several of the adjacent uncharged residues were also changed, but with relatively little effect. Terlau *et al*. [43] also measured permeation effects. Neutralization of the inner ring carboxylates dramatically reduced permeation and neutralization of the outer ring carboxylates had more modest but significant effects on permeation. See also the extensive studies of Schlief *et al*. [44], Chiamvimonvat *et al*. [45], Favre *et al*. [46]. At this point, it was clear that TTX binding was influenced by multiple residues in what is certainly the outer vestibule and the narrow region controlling permeation. The demonstration that mutation of residues critically involved in permeation and selectivity greatly reduced or abolished toxin affinity established beyond any doubt that TTX and STX bind and block deep within the outer vestibule. Their interactions with multiple P loops also strongly supported the conclusion that toxin occludes the pore and interacts with residues critical for permeation and selectivity.

An important but unanswered question was the mechanism of the difference in affinity between isoforms of the Na channel. The cardiac isoform (Nav1.5) shows as much as 10^3^ lower affinity for TTX than the three brain isoforms (Nav1.1–3) and the skeletal isoform (Nav1.4). In the domain 1 P loop sequence, where the toxins were thought to bind, there are two differences between these isoforms. The Nav1.5 sequence is DCWED, but Nav1.1–4 have an aromatic residue on place of the cysteine (F for Nav1.1–3, and Y for Nav1.4) and an asparagine in place of the C-terminal aspartate. Several lines of evidence pointed to the aromatic residue as the basis of high TTX affinity for Nav1.1–4, as opposed to the aspartate, as the basis of Nav1.5 low affinity. The cardiac isoform is more sensitive to block by some divalent ions (e.g., [47,48]), which have a higher affinity for cysteine compared to aromatic residues, and these ions compete with TTX (e.g., [14]). Replacement of cysteine by tyrosine or phenylalanine in Nav1.5 increased TTX affinity almost 10^3^-fold, while replacement of the aspartate with asparagine somewhat reduced affinity instead of increasing it [47]. Comparable experiments in Nav1.4 also pointed to the Y/C site as critical for high TTX affinity in the skeletal muscle isoform [49], and comparable experiments in Nav1.2 pointed to the importance of the F/C site [50]. These studies demonstrated clearly that the cysteine-aromatic residue difference accounted for all, or almost all of the isoform difference in TTX affinity between Nav1.5 and Nav1.1–4.

A large number of residues in the vestibule clearly influenced TTX binding. In an effort to characterize these interactions, Penzotti *et al*. [51] undertook a quantitative study of change in binding and unbinding rates with mutations of each of the Nav1.4 vestibule residues known to influence TTX binding and compared them with STX, yielding ΔΔG values for the different mutations. The experimentally determined effects of outer and inner ring mutations on IC_50_, k_on_ and k_off_ of TTX block are compared in Table 1. The most important residue of the inner ring in determining the efficacy of TTX block was Glu-755 (Nav1.4 numbering), with the rank order of importance of the different domain residues II ≫ III> I. The ΔΔG’s for neutralizations D400A, E755A, and K1237A from domains I, II, and III were 3.3, 5.4, and 4.1 kcal/mol (see Table 2). Reductions in k_on_’s were primarily responsible for the decreases in affinity. However, E755A did show a small increase in k_off_. The relative importance of the domains was different for the outer ring. The rank order for neutralization of outer ring residues of the domains was I ≫ II> IV, suggesting that Glu-403 of domain I was of major importance to TTX affinity. E403Q showed a ΔΔG of 5.2 kcal/mol, with ΔΔG’s of E758Q and D1532N smaller at 3.3 and 2.4 kcal/mol (Table 2). The changes in K_d_ were entirely associated with decreases in k_on_.

The effects of mutations were not simply the removal of negative charges. M1240K in domain III resulted in a 5.4 kcal/mol ΔΔG, while M1240E reduced affinity, instead of increasing it, by a ΔΔG of 4.8 kcal/mol. Again the major cause in reduction in affinity was a decrease in k_on_. As expected, mutations at the Tyr-401 site reduced affinities of both STX and TTX, with greater effect on TTX. The Y/C mutation resulted in affinity similar to that in human Nav1.5, which normally contains a Cys at this site.

Several aspects of this study were noteworthy. First, ΔΔG’s for mutations of selectivity filter residues were large. Second, the outer ring carboxylate neutralizations produced similar and very large ΔΔG’s, Third, the changes resulted almost entirely from changes in k_on_, with little change in k_off_. The Y/C mutation had a much larger ΔΔG for TTX than for STX.

## 6. Modeling the TTX Binding Site

Since TTX binding was a guide to the regions of the protein sequence that composed the outer vestibule and selectivity region, modeling of TTX interactions has been very helpful to gaining insight into both channel structure and TTX binding. Following the argument that TTX is a rigid molecule and binds to multiple sites in the outer vestibule, Lipkind and Fozzard [52] developed a molecular model of the outer vestibule, using the logic of lock and key. They formed hairpin P loops from the sequences in each domain with the pore-facing segment arranged as β-strands. This, for example, meant that in the domain I sequence the outer ring glutamate and the cysteine/tyrosine/phenylalanine would face into the pore, as well as the inner ring aspartate because it is part of the turn (see Figure 1). The four P loops with the two rings of charged residues were docked symmetrically onto the TTX and STX molecules to form a funnel-shaped binding site. A critical feature of this outer vestibule model was the requirement of clockwise organization of the four P loops, when viewed from the outside. This clockwise pattern has subsequently been supported by several studies of binding by asymmetrical toxins [53,54].

MacKinnon and colleagues described at 3.2 Å resolution the X-ray structure of a bacterial K channel called KcsA (minus its N- and C-terminae) [55]. Although this channel is not voltage dependent, it has the same selectivity properties of the voltage-gated channel pore. It contains two membrane-spanning α-helical segments M1 and M2. Four subunits combine to form a symmetrical permeation path, lined by a “teepee” of converging M2 transmembrane helices. The P loops between the four sets of paired helices, which are homologous with voltage-gated K channels, are arranged to form the 12 Å-long selectivity filter of the channel. The outer vestibule is lined by the P loop backbone carbonyls, with the side chains facing away from the pore.

The model of the S5-P-S6 Na channel pore-forming unit of Nav1.4 [56] was based on the backbone coordinates of the closed KcsA channel unit [55], along with an α-helix-turn-β-strand motif for the P loops to compose an energetically appropriate outer vestibule for binding of the guanidinium toxins and μ-conotoxin. This Na channel vestibule-selectivity region was made wider than that of KcsA in order to accommodate the large toxin molecules within the vestibule, along with the side chains of the lining residues that face the pore. Consequently, the backbone configurations of the Na channel P loops differ slightly from those of the KcsA channel. The P loop turns of the Na channel model are exactly at the DEKA selectivity filter residues, with the β-strands ascending back toward the extracellular side of the pore [56,57]. In comparison, K channel turn residues are located deeper into the pore and the selectivity filter region is distributed over about a 15 Å segment.

In our model of the Na channel outer vestibule, the P loops of domains I–IV were docked into the extracellular part of the inverted teepee formed by the C-terminal segments of S5 helices and the *N*-terminal segments of the S6 helices, which were located on the basis of the KcsA M1 and M2 main chains. As a result, each P loop formed densely packed contacts with the α-helices of S5 and S6 segments of its own domain and S6 of the neighboring domain. Assembly of the four P loop αβ-hairpins then formed the guanidinium toxin binding pocket. The retained interactions with the guanidinium toxins include the following features:

1. The 1,2,3 guanidinium group of TTX and the 7,8,9 guanidinium group of STX are directed into the pore, where they interact most strongly with Glu-755 of domain II and Asp-400 of domain I.
2. The 1,2,3 guanidinium group of STX, which is located at right angles to the plane of the other guanidinium group in the rigid STX structure, interacts with Asp-1532 of domain IV.
3. In the plane of the 1,2,3 guanidinium group on the opposite side of STX is a C-12 *gem*-diol, postulated to interact with Glu-758 of domain II.
4. There is a strong nonbonded interaction between the aromatic ring of Tyr-401 of domain I and the nonpolar surface of TTX.

## 7. Comparison of Model with Experimental Identification of Guanidinium Toxin Interactive Groups

**Figure 2:**
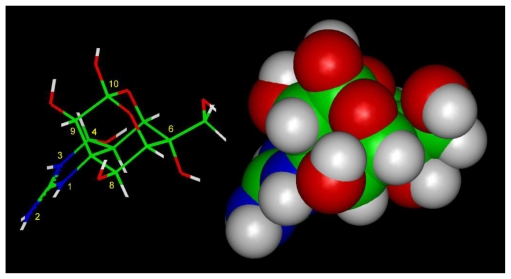
Schematic of tetrodotoxin structure (left panel) and space-filling model (right panel).

Kao and coworkers [1] identified the active groups in TTX and STX by study of structural analogs. For TTX these include the guanidinium group and the hydroxyls at C9 and C10 (Figure 2). The guanidinium group seems to form an ion-pair with an anionic site on the receptor and the C9-OH and C10-OH form hydrogen bonds at other sites [58]. The low affinity of anhydrotetrodotoxin, where C4 and C9 are joined by an oxygen bridge, supports this proposal [59,60]. 4-Epitetrodotoxin, where the -H and -OH were reversed from TTX is about half as potent. Therefore, the change in activity of anhydrotetrodotoxin can be explained by loss of the C9-OH group and its hydrogen bond to the receptor. Interestingly, the Nav1.6 isoform seems to retain its affinity for anhydroTTX [61]. Modification of both C9-OH and C10-OH in tetrodonic acid resulted in complete loss of binding [59]. 6-Epitetrodotoxin and 11-deoxytetrodotoxin (with -CH_3_ instead of -CH_2_OH) have low binding Na channel affinity (about 0.01x) [62,63] that confirms also the importance of the 11-CH_2_OH group.

**Figure 3:**
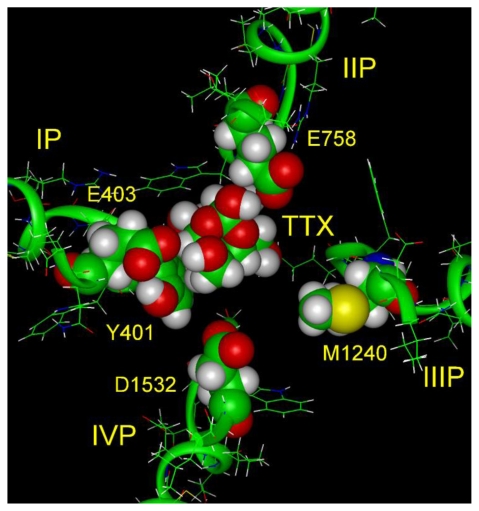
Top view of a new docking model of TTX in the outer vestibule.

**Figure 4:**
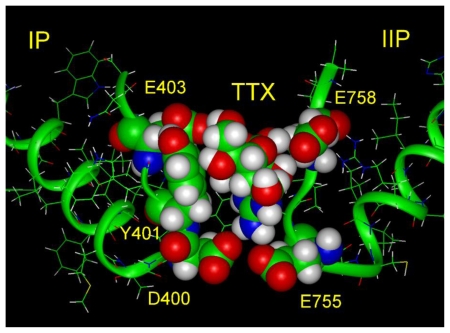
Side view of a new docking model of TTX in the outer vestibule.

Yang *et al*. [62] identified seven possible interaction sites, but a more conservative analysis suggests that only the guanidinium group and the hydroxyls at C9 and C10 (for TTX) or C12 (for STX) are the more critical sites, partly because they show remarkable stereospecificity. When the guanidinium group of TTX and the 7,8,9 guanidinium group of STX are aligned, then the hydroxyls at C9 and C10 for TTX and the two hydroxyls at C12 for STX are almost perfectly aligned [58]. Based on the argument that the 1,2,3 guanidinium of TTX and the 7,8,9 guanidinium of STX are critical, they are reserved for interaction with the DEKA selectivity filter. Using this alignment the energetical optimization of TTX in its complex with the outer vestibule has led to the structures in Figures 3 and 4 (top and side views). TTX spans the outer vestibule model between domains I and II and its 1,2,3 guanidinium group is in immediate contact with Asp-400 and Glu-755. The guanidinium group of TTX simultaneously forms hydrogen bonds with these residues.

The optimal Tyr-401 - TTX interaction occurred with the nonpolar C4-C5-C7-C8 side of the toxin by van der Waals contacts. The calculated nonbonded interaction energy change with the Y401C mutation was 4.9 kcal/mol (−6.7 kcal/mol with Tyr-401 and −1.8 kcal/mol with Cys-401). This is compared to the 4.8 kcal/mol change measured experimentally with this mutation (Table 2). The dense packing of TTX with the domain P loop would prevent sulfhydryl interactions with this Cys, as found experimentally [64]. This orientation of TTX placed the C9 and C10 hydroxyl groups near Glu-758 and the C11 hydroxyl group near Glu-403. However, only one hydroxyl formed a hydrogen bond with Glu-758, explaining the weaker interaction of TTX with this residue than for STX, whose two hydroxyls at C12 hydrated ketone simultaneously formed hydrogen bonds with Glu-758. TTX failed to make contact with Asp-1532 of domain IV, but in contrast, STX does make intimate contact. STX loss of energy of interaction with mutation of Asp-1532 was −6.2 kcal/mol, and TTX loss was only −2.4 kcal/mol (Table 2). This orientation is also supported by the results of Choudhary *et al*. [65] for binding of 1,4 gonyautoxin, which has a sulfate in the C11 position.

The calculated energies of interaction of Asp-400, Glu-755, Glu-758, and Asp-1532 with TTX were −4.0, −4.1, −3.6, and −2.2 kcal/mol. In this model the interactions with Asp-400 and Glu-755 are almost the same, while the experimental estimates show a noticeable difference: −3.3 and −5.4 kcal/mol (Table 2). However, we need to underline that the side chain of Glu-755 is important not only for interaction with TTX, but also important for the structural integrity of the selectivity filter itself. This is probably why experimental substitution of Lys-1237, which interacts with Glu-755 and maintains the selectivity ring structure, also decreased binding of TTX with the loss of about 4 kcal/mol. If Lys-1237 was acting with TTX electrostatically and the ring were rigid, we would expect that neutralization would have increased binding affinity.

Santarelli *et al*. [66] presented convincing data that TTX participates in a “cation-π-interaction” with the aromatic ring of Tyr-401 in Nav1.4, explaining the need for an aromatic residue at this position. Progressive fluorination of a benzene ring inserted in the 401 position reduced TTX binding energy linearly to a maximal change of about 2 kcal/mol. This represents about half the change when cysteine, the Nav1.5 residue, is in the 401 position. Recall that substitution of cysteine for Tyr-401 produces a 4.8 kcal/mol change. Their examination of the binding of TTX inside the outer vestibule, using the model of Lipkind and Fozzard [56], ignored possible interactions of TTX with the carboxylates of the inner and outer charge rings and focused on direct contacts of the TTX guanidinium group with the aromatic ring. As a consequence they may have overestimated the proximity of the guanidinium group of TTX to the side chain of Tyr-401. It is difficult to orient TTX within the vestibule without considering hydrogen bonds (salt bridges) between the guanidinium group of TTX and the negatively charged carboxylates Asp-400 and Glu-755 in the selectivity filter. Such interactions with the selectivity filter have the important result of preventing Na permeation. Also, perhaps the π-electron interaction [66] is an intermediate on the way to final binding. An alternative model of the outer vestibule proposed by Tikhonov and Zhorov [67] was not useful for the π-electron analysis because it did not orient the aromatic ring of Tyr-401 toward the pore.

Both the Lipkind-Fozzard and the Tikhonov-Zhorov models of TTX/STX binding assume that the outer vestibule is normally rigid. There are several pieces of experimental evidence that suggest that the outer vestibule is somewhat flexible. However, for purposes of interactions and site location, an induced fit between the toxin and the site is equally revealing. As already noted, any possible conformational change in the binding site has no significant effect on channel gating. Conformational changes during toxin binding would imply the existence of several stages in the binding process. Indeed, there are experimental results indicating that several stages in TTX/STX binding do occur [51,68]. Because the toxin molecule is not symmetrical, it is crucial that it be properly oriented before it can be tightly bound. The initial attraction to the pore could be significantly influenced by the pore’s negative field [69], and the toxin orientation would be strongly influenced by the charged residues as it entered the vestibule. The finding that neutralization of the charged vestibule residues reduced affinity by reducing k_on_, rather than increasing k_off_, implies that electrostatic orientation is a major part of the carboxylate contribution to binding orientation [18,51], positioning the toxin so that close interactions such as hydrogen bonding could occur. And it is true for both tonic and use-dependent block by TTX. Mutations of the negatively charged residues of the outer ring [18] and different levels of fluorinations of Try-401 [66] have produced the same relative changes in binding affinities as for tonic and stimulated states. Therefore, both states, despite any conformational changes, involve the same physical interactions between TTX and the outer vestibule of the Na channel.

## 8. Summary

The guanidinium toxins bind with high affinity deeply within the voltage-gated Na channel’s outer vestibule through multiple interactions to carboxylates and other residues. The binding is selective for voltage-gated Na channels, and this property has made radio labeled toxin crucial in the channel’s cloning. Binding produces complete block by interaction with the selectivity filter, while largely occluding the pore. Some isoforms have lower affinity. The best known example of this is the lower affinity for the cardiac Na channel, and this results from a cysteine just above the selectivity filter in that isoform, instead of an aromatic residue. The compact and rigid toxin structure has assisted molecular modeling of the channel’s outer vestibule, providing insight into the channel’s permeation and selectivity mechanisms. The toxins may eventually assist in direct structural studies of the voltage-gated Na channel.

## Figures and Tables

**Table 1:** Changes in IC_50_, k_on_, and k_off_ for TTX with mutations in the outer vestibule (modified from Penzotti *et al*. [51]).

Mutation	Equilibrium IC_50_(μM)	k_on_ (M^−1^s^−1^)	k_off_ (s^−1^)	IC_50_ ratio

Native Na_v_1.4	0.036 ± 0.006	3.53 × 10^5^	1.02 × 10^−2^.	1
D400A	6.0 ± 0.7	2.93 × 10^2^	1.26 × 10^−2^	168
E403Q	161 ± 14	2.48 × 10^2^	1.57 × 10^−2^	4521
E755A	229 ± 21	2.90 × 10^2^	2.69 × 10^−2^	6412
E758Q	6.2 ± 0.3	4.86 × 10^3^	1.47 × 10^−2^	174
K1237A	23 ± 2	1.62 × 10^2^	1.49 × 10^−2^	641
M1240E	87 ± 5	4.15 × 10^2^	1.98 × 10^−2^	2433
M1240K	238 ± 17	1.02 × 10^2^	1.58 × 10^−2^	6678
D1532N	1.5 ± 0.2	6.14 × 10^2^	1.45 × 10^−2^	43

**Table 2:** Changes in equilibrium binding free energy of STX and TTX for the outer vestibule mutants (From Penzotti *et al*. [51]).

Mutation	ΔΔG_STX_ ± SE	ΔΔG_TTX_ ± SE	ΔΔG_STX_/ΔΔG_TTX_ ± SE

D400A	3.8 ± 0.1	3.3 ± 0.3	1.2 ± 0.1
Y401D	2.4 ± 0.1	5.2 ± 0.3	0.5 ± 0.2
Y401C	2.7 ± 0.1	4.8 ± 0.3	0.6 ± 0.1
E403Q	6.2 ± 0.1	5.2 ± 0.3	1.2 ± 0.05
E755A	5.7 ± 0.1	5.4 ± 0.3	1.1 ± 0.1
E758Q	6.0 ± 0.1	3.3 ± 0.3	1.8 ± 0.05
K1237A	3.9 ± 0.2	4.1 ± 0.3	1.0 ± 0.1
M1240E	3.9 ± 0.1	4.8 ± 0.3	0.8 ± 0.1
M1240K	6.1 ± 0.2	5.4 ± 0.3	1.1 ± 0.1
D1532N	6.2 ± 0.2	2.4 ± 0.3	2.6 ± 0.1

## References

[1] Kao CY Tetrodotoxin, saxitoxin and their significance in the study of excitation phenomenon Pharm Rev 1966 18 997 1049 5328391

[2] Kao CY Levinson SR Tetrodotoxin, saxitoxin, and the molecular biology of the sodium channel Ann NY Acad Sci 1986 479 1 443 2433985

[3] Narahashi T Deguchi T Urakawa N Ohkubo Y Stabilization and rectification of muscle fiber membrane by tetrodotoxin Amer J Physiol 1960 198 934 938 14426011 10.1152/ajplegacy.1960.198.5.934

[4] Narahashi T Moore JW Scott W Tetrodotoxin blockage of sodium conductance increase in excitation J Gen Physiol 1964 47 965 974 14155438 10.1085/jgp.47.5.965 PMC2195365

[5] Hille B Pharmacological modifications of the sodium channels of frog nerve J Gen Physiol 1968 51 199 219 5641635 10.1085/jgp.51.2.199 PMC2201123

[6] Hille B Ion Channels of Excitable Membranes Sinauer Associates, Inc Sunderland, MA, USA 2001 534

[7] Kao CY Nishiyama A Actions of saxitoxin on peripheral neuromuscular systems J Physiol 1965 180 50 66 5860225 PMC1357367

[8] Ulbricht W Wagner HH The influence of pH on equilibrium effects of tetrodotoxin on myelinated nerve fibres of *Rana esculenta* J Physiol 1975 252 159 184 492 10.1113/jphysiol.1975.sp011139 PMC1348473

[9] Ulbricht W Wagner HH The influence of pH on the rate of tetrodotoxin action of myelinated nerve fibres J Physiol 1975 252 185 202 493 10.1113/jphysiol.1975.sp011140 PMC1348474

[10] Reed JK Raftery MA Properties of the tetrodotoxin binding component in plasma membranes isolated from *Electrophorus electricus* Biochemistry 1976 15 944 953 3213 10.1021/bi00650a002

[11] Spalding BC Properties of toxin-resistant sodium channels produced by chemical modification in frog skeletal muscle J Physiol 1980 305 485 500 6255148 10.1113/jphysiol.1980.sp013377 PMC1282986

[12] Worley JF III French RJ Krueger BK Trimethyloxonium modification of single batrachotoxin-activated sodium channels in planar bilayers J Gen Physiol 1986 87 327 349 2419487 10.1085/jgp.87.2.327 PMC2217599

[13] Henderson R Ritchie JM Strichartz GR Evidence that tetrodotoxin and saxitoxin act at a metal cation binding site in the sodium channels of nerve membrane Proc Natl Acad Sci USA 1974 71 3936 3940 4530274 10.1073/pnas.71.10.3936 PMC434301

[14] Doyle DD Guo Y Lustig SL Satin J Rogart RB Fozzard HA Divalent cation competition with [3H]saxitoxin binding to tetrodotoxin-resistant and -sensitive sodium channels J Gen Physiol 1993 101 153 182 8384241 10.1085/jgp.101.2.153 PMC2216764

[15] Aggarwal R Shorofsky SR Goldman L Balke CW Tetrodotoxin-blockable calcium currents in rat ventricular myocytes: a third type of cardiac cell sodium current J Physiol 1997 505 353 369 9423179 10.1111/j.1469-7793.1997.353bb.x PMC1160070

[16] Sha Q Robinson SW McCulle SL Shorofsky SR Welling PA Goldman L Balke CW An antisense oligonucleotide against H1 inhibits the classical sodium current but not I_Ca(TTX)_ in rat ventricular cells J Physiol 2003 547 435 440 12562928 10.1113/jphysiol.2002.035246 PMC2342645

[17] Sun H Varela D Chartier D Ruben PC Nattel S Zamponi GW LeBlanc N Differential interactions of Na channel toxins with T-type Ca channels J Gen Physiol 2008 132 101 113 18591418 10.1085/jgp.200709883 PMC2442173

[18] Boccaccio A Moran O Imoto K Conti F Tonic and phasic tetrodotoxin block of sodium channels with point mutations in the outer pore region Biophysical J 1999 77 229 240 10.1016/S0006-3495(99)76884-0 PMC1300324 10388752

[19] Sheets MF Hanck DA Molecular action of lidocaine on the voltage sensors of sodium channels J Gen Physiol 2003 121 163 175 12566542 10.1085/jgp.20028651 PMC2217326

[20] Bezanilla F The voltage sensor in voltage-dependent ion channels Physiol Rev 2000 80 555 592 10747201 10.1152/physrev.2000.80.2.555

[21] Armstrong CM Bezanilla F Charge movement associated with the opening and closing of the activation gates of the Na channel J Gen Physiol 1974 63 533 552 4824995 10.1085/jgp.63.5.533 PMC2203568

[22] Heggeness ST Starkus JG Saxitoxin and tetrodotoxin. Electrostatic effects on sodium channel gating current in crayfish axons Biophys J 1986 49 629 643 2421792 10.1016/S0006-3495(86)83690-6 PMC1329510

[23] Satin J Limbertis JT Kyle JW Rogart RB Fozzard HA The saxitoxin-tetrodotoxin binding site on cloned rat brain Iia Na channels is in the transmembrane electrical field Biophys J 1994 67 1007 1014 7811911 10.1016/S0006-3495(94)80566-1 PMC1225453

[24] Moczydlowski E Hall S Garber SS Strichartz GS Miller C Voltage-dependent blockade of muscle Na channels by guanidinium toxins J Gen Physiol 1984 84 687 704 6096479 10.1085/jgp.84.5.687 PMC2228759

[25] French RJ Worley JF III Krueger BK Voltage-dependent block by saxitoxin of sodium channels incorporated, to planar lipid bilayers Biophys J 1984 45 301 310 6324910 10.1016/S0006-3495(84)84156-9 PMC1435261

[26] Khan A Romantseva L Lam A Lipkind GM Fozzard HA Role of the outer ring carboxylates of the rat skeletal muscle sodium channel pore in proton block J Physiol 2002 543 71 85 12181282 10.1113/jphysiol.2002.021014 PMC2290475

[27] Ritchie JM Rogart RB The binding of saxitoxin and tetrodotoxin to excitable tissue Rev Physiol Biochem Pharmacol 1977 79 1 50 335473 10.1007/BFb0037088

[28] Agnew WS Levinson SR Brabson JS Raftery MA Purification of the tetrodotoxin-binding component associated with the voltage-sensitive sodium channel from *Electrophorus electricus* electroplax membranes Proc Natl Acad Sci USA 1978 75 2606 2610 275831 10.1073/pnas.75.6.2606 PMC392611

[29] Noda M Shimizu S Tanabe T Takai T Kayano T Ikada H Takahashi H Nakayama H Kanaoka Y Minamino N Kangawa K Matsuo H Raftery MA Hirose T Furutani Y Inayama S Hayashida H Miyata T Numa S Primary structure of *Electrophorus electricus* sodium channel deduced from cDNA sequence Nature (Lond) 1984 312 121 127 6209577 10.1038/312121a0

[30] Trimmer JS Cooperman SS Tomiko SA Zhou J Crean SM Boyle MB Kallen RG Sheng Z Barchi RL Sigworth FJ Goodman RH Agnew WS Mandel G Primary structure and functional expression of a mammalian skeletal muscle sodium channel Neuron 1989 3 33 49 2559760 10.1016/0896-6273(89)90113-x

[31] George AL Jr Ledbetter DH Kallen RG Barchi RL Assignment of a human skeletal muscle sodium channel alpha-subunit gene (SCN4aA) to 17q23.–25.3 Genomics 1991 9 555 556 1851726 10.1016/0888-7543(91)90425-e

[32] Rogart RB Cribbs LL Muglia LK Kephart DD Kaiser MW Molecular cloning of a putative tetrodotoxin-resistant rat heart Na channel isoform Proc Natl Acad Sci USA 1989 86 8170 8174 2554302 10.1073/pnas.86.20.8170 PMC298237

[33] Gellens ME George AL Jr Chen L Chahine M Horn R Barchi RL Kallen RG Primary structure and functional expression of the human cardiac, voltage-dependent sodium channel Proc Natl Acad Sci USA 1992 89 554 558 1309946 10.1073/pnas.89.2.554 PMC48277

[34] Goldin AL Evolution of voltage-gated Na channels J Exp Biol 2002 205 575 584 11907047 10.1242/jeb.205.5.575

[35] Creighton TE Proteins WH Freeman and Company New York, NY, USA 1984

[36] Guy HR Conti F Pursuing the structure and function of voltage-gated channels Trends Neurosci 1990 13 201 206 1694324 10.1016/0166-2236(90)90160-c

[37] Guy HR Seetharamulu P Molecular model of the action potential sodium channel Proc Natl Acad Sci USA 1986 83 508 512 2417247 10.1073/pnas.83.2.508 PMC322889

[38] Hille B The permeability of the sodium channel to metal ions in myelinated nerve J Gen Physiol 1971 58 599 619 5315827 10.1085/jgp.58.6.599 PMC2226049

[39] Sigworth FJ Spalding BC Chemical modification reduces the conductance of sodium channels in nerve Nature 1980 283 293 295 6965422 10.1038/283293a0

[40] Guy HR A model relating the sodium channel’s structure to its function Molecular Biology of Ionic Channels Agnew WS Claudio T Sigworth FJ Academic Press San Diego, CA, USA 1988 33 289 308

[41] Pusch M Noda M Stühmer W Numa S Conti F Single point mutations of the sodium channel drastically reduce the pore permeability without preventing its gating Eur Biophys J 1991 20 127 133 1660394 10.1007/BF01561134

[42] Noda M Suzuki H Numa S Stühmer W A single point mutation confers tetrodotoxin and saxitoxin insensitivity on the sodium channel II FEBS Lett 1989 259 213 216 2557243 10.1016/0014-5793(89)81531-5

[43] Terlau H Heinemann SH Stühmer W Pusch M Conti F Imoto K Numa S Mapping the site of block by tetrodotoxin and saxitoxin of sodium channel II FEBS Letts 1991 293 93 96 1660007 10.1016/0014-5793(91)81159-6

[44] Schleif T Schonherr R Imoto K Heinemann SH Pore properties of rat brain II sodium channels mutated in the selectivity filter domain Eur Biophys J 1996 25 75 91 9035373 10.1007/s002490050020

[45] Chiamvimonvat N Perez-Garcia MT Tomaselli GF Marban E Control of ion flux and selectivity by negatively charged residues in the outer mouth of rat sodium channels J Physiol 1996 491 51 59 9011621 10.1113/jphysiol.1996.sp021195 PMC1158758

[46] Favre I Moczydlowski E Schild L On the structural basis for ionic selectivity among Na, K, and Ca in the voltage-gated sodium channel Biophys J 1996 71 3110 3125 8968582 10.1016/S0006-3495(96)79505-X PMC1233800

[47] Satin J Kyle JW Chen M Bell P Cribbs LL Fozzard HA Rogart RB A mutant of TTX-resistant cardiac sodium channels with TTX-sensitive properties Science 1992 256 1202 1205 1375397 10.1126/science.256.5060.1202

[48] Favre I Moczydlowski E Schild L Specificity for block by saxitoxin and divalent cations at a residue which determines sensitivity of sodium channel subtypes to guanidinium toxins J Gen Physiol 1995 106 203 229 8537816 10.1085/jgp.106.2.203 PMC2229260

[49] Backx PH Yue DT Lawrence JH Marban E Tomaselli GF Molecular localization of an ion-binding site within the pore of mammalian sodium channels Science 1992 257 248 251 1321496 10.1126/science.1321496

[50] Heinemann SH Terlau H Imoto K Molecular basis for pharmacological differences between brain and cardiac channels Eur J Physiol 1992 422 90 92 10.1007/BF00381519 1331981

[51] Penzotti JL Fozzard HA Lipkind GM Dudley SC Jr Differences in saxitoxin and tetrodotoxin binding revealed by mutagenesis of the Na channel outer vestibule Biophys J 1998 75 2647 2657 9826589 10.1016/S0006-3495(98)77710-0 PMC1299940

[52] Lipkind GM Fozzard HA A structural model of the tetrodotoxin and saxitoxin binding site of the Na channel Biophys J 1994 66 1 13 8130328 10.1016/S0006-3495(94)80746-5 PMC1275657

[53] Dudley SC Jr Chang N Hall J Lipkind GM Fozzard HA French RJ μ-Conotoxin GmA interactions with the voltage-gated Na channel predict a clockwise arrangement of the domains J Gen Physiol 2000 116 679 690 11055996 10.1085/jgp.116.5.679 PMC2229485

[54] Li RA Ennis IL French RJ Dudley SC Jr Tomaselli GF Marban E Clockwise domain arrangement of the sodium channel revealed byμ-conotoxin (GmA) docking orientation J Biol Chem 2001 276 11072 11077 11154701 10.1074/jbc.M010862200

[55] Doyle DA Cabral JM Pfuetzner RA Kuo A Gulbis JM Cohen SL Chait BT MacKinnon R The structure of the potassium channel: molecular basis of K conduction and selectivity Science 1998 280 69 77 9525859 10.1126/science.280.5360.69

[56] Lipkind GM Fozzard HA KcsA crystal structure as framework for a molecular model of the Na channel pore Biochemistry 2000 40 6786 6794 10889022 10.1021/bi000486w

[57] Yamagishi T Janecki M Marban E Tomaselli GF Topology of the P segments in the sodium channel revealed by cysteine mutagenesis Biophys J 1997 73 195 204 9199784 10.1016/S0006-3495(97)78060-3 PMC1180921

[58] Kao CY Walker SE Active groups of saxitoxin and tetrodotoxin as deduced from actions of saxitoxin analogues on frog muscle and squid axon J Physiol 1982 323 619 637 6284918 10.1113/jphysiol.1982.sp014095 PMC1250379

[59] Narahashi T Moore JW Posten RN Tetrodotoxin derivatives: Chemical structure and blockage of nerve membrane conductance Science 1967 156 976 979 6023268 10.1126/science.156.3777.976

[60] Kao CY Yasumoto T Actions of 4-epitetrodotoxin and anhydrotetrodotoxin Toxicon 1985 23 725 729 2418538 10.1016/0041-0101(85)90002-9

[61] Rosker C Lohberger B Hofer D Steinecker B Quasthoff S Schreibmayer W The TTX metabolite 4,9-anhydro-TTX is a highly specific blocker of the Nav1.6 voltage-dependent sodium channel Amer J Physiol Cell Physiol 2007 293 C783 789 17522141 10.1152/ajpcell.00070.2007

[62] Yang L Kao CY Yasumoto T Actions of 6-*epi* tetrodotoxin and 1-deoxytetrodotoxin on the frog skeletal muscle fiber Toxicon 1992 30 635 643 1325687 10.1016/0041-0101(92)90857-2

[63] Yotsu-Yamashita M Sugimoto A Takai A Yasumoto T Effects of specific modifications of several hydroxyls of tetrodotoxin on its affinity to rat brain membrane J Pharmacol Exp Ther 1999 289 1688 1696 10336569

[64] Chen SF Hartmann HA Kirsch GE Cysteine mapping in the ion selectivity and toxin binding region of the cardiac Na channel pore J Membr Biol 1997 155 11 25 9002421 10.1007/s002329900154

[65] Choudhary G Shang L Li X Dudley SC Jr Energetic localization of saxitoxin in its channel binding site Biophys J 2002 83 912 919 12124273 10.1016/S0006-3495(02)75217-X PMC1302195

[66] Santarelli VP Eastwood AL Dougherty DA Horn R Ahern CA A cation-π interactiion discriminates among sodium channels that are either sensitive or resistant to tetrodotoxin block J Biol Chem 2007 282 8044 8051 17237232 10.1074/jbc.M611334200

[67] Tikhonov DB Zhorov BS Modeling P-loops domain of sodium channel: Homology with potassium channels and interaction with ligands Biophys J 2005 88 184 197 15475578 10.1529/biophysj.104.048173 PMC1304997

[68] Guo Z Uehara A Ravindran A Bryant SH Hall S Moczydlowski E Kinetic basis for insensitivity to tetrodotoxin and saxitoxin in sodium channels of canine heart and denervated skeletal muscle Biochemistry 1987 26 7346 7356 10.1021/bi00398a003 2447944

[69] McNulty MM Edgerton GB Shah RD Hanck DA Fozzard HA Lipkind GM Charge at the lidocaine binding site residue Phe-1759 affects permeation in human cardiac voltage-gated sodium channels J Physiol 2007 581 741 755 17363383 10.1113/jphysiol.2007.130161 PMC2075178

